# Chemotactic Responses of Jurkat Cells in Microfluidic Flow-Free Gradient Chambers

**DOI:** 10.3390/mi11040384

**Published:** 2020-04-04

**Authors:** Utku M. Sonmez, Adam Wood, Kyle Justus, Weijian Jiang, Fatima Syed-Picard, Philip R. LeDuc, Pawel Kalinski, Lance A. Davidson

**Affiliations:** 1 Department of Mechanical Engineering, Carnegie Mellon University, Pittsburgh, PA 15213, USA; ut.m.son@gmail.com (U.M.S.); arwood@andrew.cmu.edu (A.W.); 2 Department of Biochemistry, Stanford University, Stanford, CA 94305-5080, USA; kjustus@alumni.cmu.edu; 3 Department of Medicine and Center for Immunotherapy, Roswell Park Cancer Institute, 837CSC Building, Elm & Carlton Streets, Buffalo, NY 14263, USA; Weijian.Jiang@RoswellPark.org; 4 Department of Oral Biology and the Center for Craniofacial Regeneration, School of Dental Medicine, University of Pittsburgh, Pittsburgh, PA 15213, USA; syedpicard@pitt.edu; 5 Department of Bioengineering, Swanson School of Engineering, University of Pittsburgh, Pittsburgh, PA 15213, USA; 6 McGowan Institute for Regenerative Medicine, University of Pittsburgh, Pittsburgh, PA 15219, USA; 7 Department of Mechanical Engineering, Biomedical Engineering, Computational Biology, and Biological Sciences, Carnegie Mellon University, Pittsburgh, PA 15213, USA; 8 Department of Developmental Biology, Department of Computational and Systems Biology, School of Medicine, University of Pittsburgh, Pittsburgh, PA 15213, USA

**Keywords:** chemotaxis, microfluidics, Jurkat cells, microfabrication, concentration gradient

## Abstract

Gradients of soluble molecules coordinate cellular communication in a diverse range of multicellular systems. Chemokine-driven chemotaxis is a key orchestrator of cell movement during organ development, immune response and cancer progression. Chemotaxis assays capable of examining cell responses to different chemokines in the context of various extracellular matrices will be crucial to characterize directed cell motion in conditions which mimic whole tissue conditions. Here, a microfluidic device which can generate different chemokine patterns in flow-free gradient chambers while controlling surface extracellular matrix (ECM) to study chemotaxis either at the population level or at the single cell level with high resolution imaging is presented. The device is produced by combining additive manufacturing (AM) and soft lithography. Generation of concentration gradients in the device were simulated and experimentally validated. Then, stable gradients were applied to modulate chemotaxis and chemokinetic response of Jurkat cells as a model for T lymphocyte motility. Live imaging of the gradient chambers allowed to track and quantify Jurkat cell migration patterns. Using this system, it has been found that the strength of the chemotactic response of Jurkat cells to CXCL12 gradient was reduced by increasing surface fibronectin in a dose-dependent manner. The chemotaxis of the Jurkat cells was also found to be governed not only by the CXCL12 gradient but also by the average CXCL12 concentration. Distinct migratory behaviors in response to chemokine gradients in different contexts may be physiologically relevant for shaping the host immune response and may serve to optimize the targeting and accumulation of immune cells to the inflammation site. Our approach demonstrates the feasibility of using a flow-free gradient chamber for evaluating cross-regulation of cell motility by multiple factors in different biologic processes.

## 1. Introduction

In nature, gradients of soluble molecules guide cellular responses in biologic activities ranging from embryogenesis [1], tissue regeneration [2] and axon growth in neural development [3] to immune cell activation and migration [4]. In addition, many immunodeficiency diseases, autoimmune diseases [5], chronic inflammation [6] and cancer metastases [7,8] develop as a result of pathological sensing or establishing inappropriate gradients of soluble signaling molecules [9]. Therefore, the characterization of cellular responses to soluble cell-signaling molecules and to their gradients is of great importance for understanding how multicellular collective behaviors are organized and regulated. Understanding the roles of gradient sensing in disease also guides us toward novel therapies including regenerative stem cell therapy for neural injury, localized drug delivery for immunomodulation of inflammatory diseases [10] and T-cell engineering for the immunotherapy of cancer [11].

Most in vitro and ex vivo migration models used to evaluate the responses of different cells to individual chemokines involve specialized migration chambers such as the Boyden chamber [12]. Boyden chambers have two reservoirs, an upper and lower chamber, separated by a semipermeable membrane. Once a chemokine is diluted into the upper chamber a transient concentration gradient develops. If responsive, cells initially seeded in the upper chamber sense the gradient and migrate into the lower chamber. Cell movements in the Boyden chamber are essentially 1-dimensional as cells move from one compartment to another. The major limitation of Boyden chambers and other similar systems lies in their inability to track spatial and temporal parameters of cell migration. Additionally, since these systems allow only limited control over the microenvironment, cells cannot be challenged by sequential events, such as the ability on one chemokine to sensitize cells to exposure by subsequent factors.

Microtechnology provides novel tools that allow precise spatiotemporal control over microenvironments to better understand the operation of complex biologic systems [13,14,15]. Advances in microfabrication over the last two decades have enabled the development of sophisticated microfluidic chemotaxis systems where dynamic and complex movement of chemotactic cells can be studied in precisely defined microenvironments [16]. The first generation of microfluidic chemotaxis systems that enabled precise control of biochemical concentration gradients relied on diffusion between adjacent laminar flow streams [17,18]. Variations of these flow-based gradient systems have been widely applied to study cellular responses to ligand gradients; however, it is now apparent that fluid flow over migrating cells can both modulate the concentration distribution of soluble molecules across the cell membrane [19] as well as exert shear stress on cells [20,21]. These coupled characteristics can cause issues as cells that normally do not experience shear stress in their native environment respond differentially to signaling molecules when shear stress is present [22]. For instance, some chemokine receptors such as CXCR4 (receptor of CXCL12) can be downregulated by laminar shear stress [23]. Moreover, bulk fluid flow continuously elutes autocrine and paracrine factors secreted by migrating cells. Continuous elution in these systems may eliminate the biochemical communication networks established by collectively migrating cells under normal physiological conditions. These studies suggest it that it is important to minimize using flow-based devices for chemotaxis studies of cells that do not normally experience flow in their physiological microenvironment.

To establish flow-free conditions, microfluidic devices can incorporate porous materials such as hydrogels, Matrigel or agarose [24,25,26,27,28,29]. Although, these systems can create stable gradients, their applications have been limited since they often require extended times to establish a gradient due to the relatively low diffusivity in porous materials [30]. More complex designs that rely on sink and source channels connected with ladder-shaped microgroove arrays have been developed to overcome this problem [31,32,33,34]. In some of these designs, transient gradients can be established [35] in a short period of time; however, fabrication of these multi-depth [36] and multilayer channels [37] can be complex and require extensive usage of cleanroom facilities [38]. However, simpler implementations of these ladder-shaped designs are possible by creating a direct pressure balance between the flow fields in sink and source channels [39,40,41], but these designs are often susceptible to mechanical noise from syringe pumps that introduce pressure imbalance across the gradient chamber [42].

We developed a microtechnology that would enable rapid progress in future studies of chemotaxis [43] through a defined set of design criteria. Such technology should (1) have the ability to generate concentration gradients in flow-free conditions; (2) have the ability to create combinatorial chemical gradients to mimic physiologically relevant complex gradient patterns; (3) be compatible with media and thin cover slips to enable high-resolution time-lapse imaging with oil immersion objectives; (4) have the ability to develop stable gradients and to exchange media to allow for long-term analysis; (5) be compatible with manual micropipettes to allow simple assembly and fluid-handling; (6) allow co-fabrication of multiple parallel chambers with different gradient slopes in a single microfluidic device; and (7) have low volume media consumption to reduce costs of reagent usage.

In this paper, we develop a microfluidic chemotaxis system to meet all of these design considerations. Our device generates flow-free concentration gradient by the diffusion of soluble species from two flow channels into a microchamber [42]. We fabricate our system by combining AM with conventional soft lithography [44] for rapid-prototyping as improvements in the feature resolution of AM techniques have facilitated the fabrication of complex microfluidic systems without a need for expensive materials and cleanroom facilities [45,46]. Our approach enables high resolution confocal imaging of diffusible fluorescent reporters and allows us to validate the gradient formation against theoretical calculations and finite-element modeling (FEM). We then apply a CXCL12 chemokine gradient together with a controlled fibronectin surface concentration to investigate Jurkat cell motility, which is a human T cell lymphoma cell line frequently used for studying cell signaling in immune cell chemotaxis [47,48].

## 2. Materials and Methods

### 2.1. System Design

**Figure 1:**
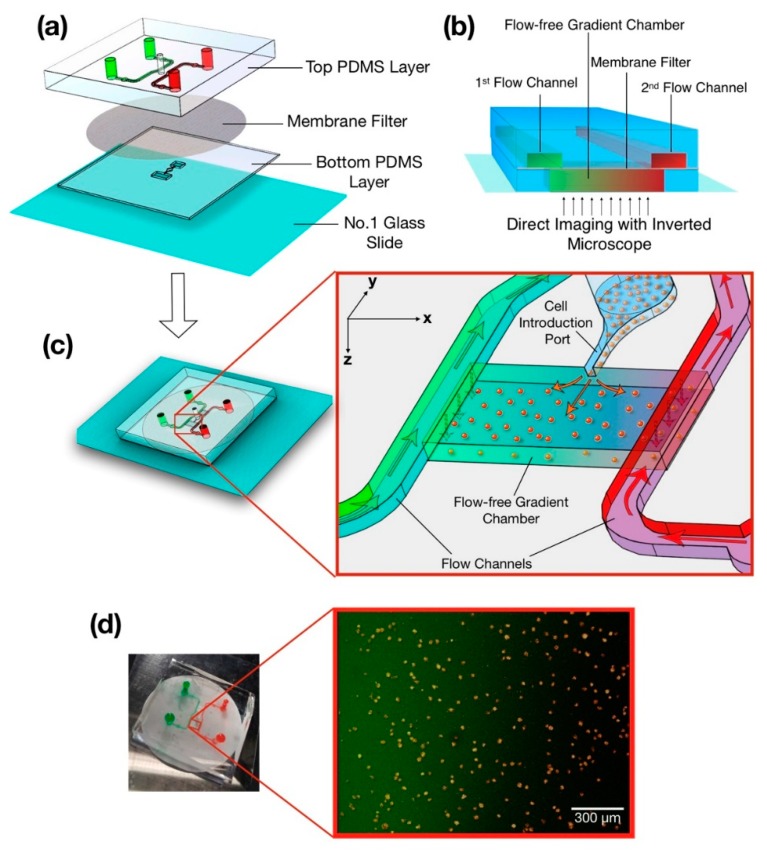
Chemotaxis of Jurkat cells in microfluidic flow-free gradient chambers. (**a**) An enlarged view of microfluidic flow-free gradient generator. The top PDMS (polydimethylsiloxane) layer had two parallel flow channels and a cell introduction port that was connected to bottom PDMS layer with a membrane filter. The entire assembly was attached to a thin glass coverslip at the bottom to enable high numerical aperture imaging. (**b**) Schematic of the flow-free gradient chamber approach. Soluble species diffused through the membrane to the bottom layer creating linear concentration gradients in the flow-free gradient chambers. (**c**) Schematic of the microfluidic device after assembly. (**inset**) The introduction of cells into the gradient chamber through the cell introduction port and the generation of a gradient across the cells. (**d**) Image of the actual microfluidic device where flow channels were filled with dye for visualization after fabrication. (**inset**) Composite fluorescent image taken from the flow-free-gradient chamber where the Jurkat cells were in a linear concentration gradient of FITC-Dextran.

The microfluidic system was made of two separate PDMS layers that sandwich a 0.4 µm PC membrane filter (EMD Millipore, Billerica, MA) (Figure 1a) [49]. The upper layer consisted of two symmetric flow channels, their respective inlet and outlet ports and a cell introduction port. The lower layer consisted of narrow and wide flow-free chambers that spanned 1.9 and 2.9 mm, respectively. Fluidic resistance was created by the membrane at the sides of the upper surface of the flow-free chamber that blocked the fluid flow from the upper flow channels to the lower chambers, leaving diffusion as the main mechanism for mass transfer between the two flow channels. Linear concentration gradients of soluble species were formed within the flow-free chamber as species diffuse through the semipermeable membrane. Continuous exchange between the upper and lower layers kept the reagent concentrations constant in the flow-free gradient chambers (Figure 1b). The concentration gradients generated within the chambers were determined based on the steady-state version of Fick’s Second Law (i.e., ∂c/∂t= 0):

(1) $$\frac{\partial^{2}c}{\partial x^{2}}+\frac{\partial^{2}c}{\partial y^{2}}+\frac{\partial^{2}c}{\partial z^{2}}=0$$

where c was the local concentration and x, y and z were Cartesian coordinates defined within the chambers (Figure 1c inset). As the concentrations at the chamber boundaries did not change in the y-direction (along the length of the chamber), the second term of Equation (1) was neglected. Although, the concentration gradient generated in x-direction (across the width of the chamber) dominates, a shallow concentration gradient was also formed in the z-direction (along the depth of the chamber) due to the height of the gradient chambers. Therefore, the height of the gradient chamber was selected carefully to avoid deviation of the concentration gradient by more than ± 5% between the top and bottom surface of the gradient chambers. Different gradient patterns can be produced depending on the application’s requirements, for instance, one flow channel can be filled with only a buffer solution while the other one can be supplied with a buffer containing a soluble molecule to create single species concentration gradients from one side. Alternatively, both channels can be filled with the solutions of different soluble molecules to generate combinatorial gradients. Furthermore, both flow channels can contain the same reagent to establish combinatorial gradients of two species on top of the uniform concentration background of a third species. Concentration gradients in the flow-free chamber can be measured directly by imaging fluorophores with similar molecular weight to that of the chemokines (e.g., FITC-Dextran MW:10,000 or Fluoro-Ruby MW: 10,000).

Small media reservoirs (500 µm radius) were fabricated in our PDMS device near the flow channel inlets to reduce flow fluctuations such as those caused by a syringe pump that may otherwise introduce a pressure imbalance between two flow channels. Cells were seeded into the gradient chambers through a cell introduction port through micropipetting prior to establishing the gradient (Figure 1c). The height of the cell introduction port was one-half of the height of the gradient chamber to generate a more homogenous distribution of cells within the gradient chamber (Figure 1c inset). Stable gradients were established once cells attached to the bottom glass surface of the gradient chamber.

### 2.2. Fabrication

**Figure 2:**
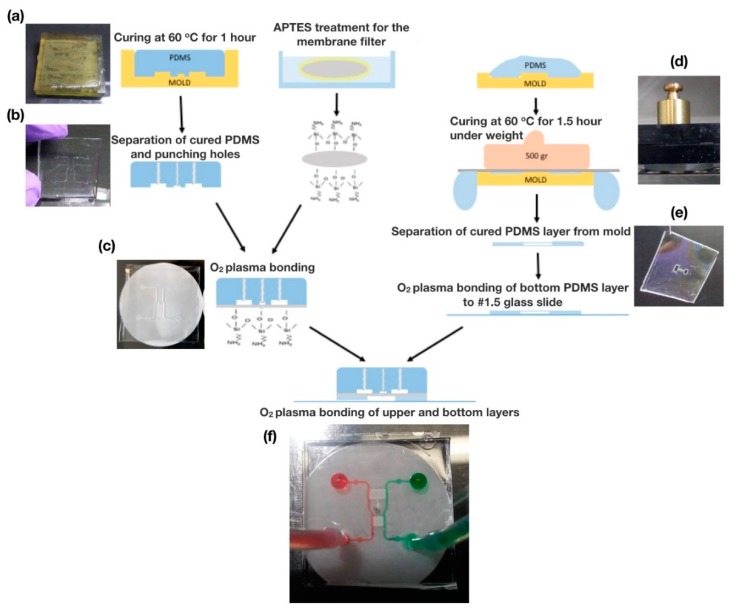
Fabrication of our flow-free microfluidic device. (**a**) Pre-cured PDMS was poured into 3D printed molds. (**b**) After curing, the top PDMS layer was separated from the mold, and inlet and outlet holes were punched. (**c**) A polycarbonate membrane was bonded to the top PDMS layer through O_2_ plasma bonding after 3-aminopropyltriethoxysilane (APTES) treatment. (**d**) A weight was placed on top of the pre-cured PDMS during the curing process to create a thin PDMS layer with the gradient chambers. (**e**) Image of the bottom PDMS layer with hollow gradient chambers after being separated from the mold. (**f**) Functional flow-free microfluidic device with green and red dye in water for flow visualization.

Both layers of the microfluidic device were fabricated using PDMS replica molding. First, negatives of the 3D channel geometries were drawn in SolidWorks 2016 (Dassault Systèmes, Vélizy-Villacoublay, France). The negative molds for both layers of the device were manufactured using VIPER si2T Stereolithography System (3D Systems, Rock Hill, SC, USA) in high quality mode with Accura SI 10 Polymer as the photocurable resin. Fabricated molds were post-cured with UV and their functional surfaces were coated with Tridecafluoro-1,1,2,2-tetrahydrooctyl-1-trichlorosilane (TFOCS, United Chemical Technology, product no. T2492, Horsham, PA, USA). Sylgard 184 PDMS (Dow Corning, Midland, MI, USA), was mixed with its curing agent at a 5:1 mass ratio, and the mixture was degassed. Pre-cured PDMS was then poured on the molds slowly to avoid any bubble formation (Figure 2a). For the fabrication of the thin lower layer, a polycarbonate (PC) sheet was laid over the pre-cured PDMS and a 500-g weight was placed on top of the PC sheet to put mechanical pressure on the pre-cured PDMS to reduce the final PDMS thickness needed for hollow gradient chambers (Figure 2d,e). Upper and lower parts were then placed in a 60 °C convection oven for 1 hour. After curing, the lower part was removed from the mold and bonded to a 45 mm × 50 mm #1.5 cover slip after treating their surfaces with oxygen plasma (Harrick Plasma Cleaner, 1 min, 18 W). The upper layer was also removed from the mold, and its inlet and outlet holes were punched with a G20 blunt tip needle (Figure 2b). Integration of the membrane filter and the PDMS parts was accomplished through a silane coupling technique, which was performed by immersing the PC membrane filter in a 5% solution of 3-aminopropyltriethoxysilane (APTES, Sigma–Aldrich, St. Louis, MO, USA) that has been pre-warmed to 80 °C for 20 min. The solution was covered during the process to avoid evaporation. Later, the membrane filter was removed from the solution and both faces were dried for 10 s. Immediately afterwards, the membrane filter and the upper PDMS layer of the microfluidic system were placed in an oxygen plasma cleaner for 20 s to create an irreversible bond between them (Figure 2c). The lower PDMS–glass slide assembly was bound to the upper PDMS layer in the same manner so that the PC membrane was sandwiched between these two PDMS layers. To improve alignment, we assembled the device within the field-of-view of a stereo microscope immediately after the oxygen plasma treatment (see Figure 2f for assembled device).

Before using the microfluidic system in cell migration studies, the gradient chambers were coated with fibronectin, which is one of the most commonly used extracellular matrix (ECM) proteins for in vitro chemotaxis assays [50]. The fibronectin was diluted to the desired concentration in 0.2 µm filtered deionized (DI) water, chambers filled with fibronectin solution through the cell introduction port, and the microfluidic system kept at 37 °C until the solution dried.

### 2.3. Experimental Setup

Our microfluidic system was connected to a Chemyx Fusion digital double-syringe pump (Thermo Scientific, Waltham, MA, USA) with 1 ml plastic syringes (BD plastic, Franklin Lakes, NJ, USA) and thin polyethylene tubing (ID: 0.58 mm, OD:0.965 mm) to minimize reagent consumption. The microfluidic system was then placed in a stage-top environmental chamber (Live Cell; Pathology Devices, Westminster, MD, USA) where temperature, humidity, and CO_2_ concentration was controlled by an environmental chamber control unit to maintain incubation conditions (37 °C, 5% CO_2_, and %90 humidity). 

Confocal time-lapse sequences for cell tracking and gradient observation were captured using a confocal laser scan head (SP5 Leica Microsystems, Wetzlar, Germany) mounted on an inverted compound microscope (DMI6000, Leica Microsystems) equipped with APO 10×/0.4 NA dry objective. A 488 nm Argon laser was used to illuminate stained cells and FITC-Dextran, and a 543 nm HeNe laser was used for live gradient observation (Fluoro-Ruby, Merck Millipore, Burlington, MA, USA). High resolution images of Jurkat cells were acquired using APO 63×/1.4 NA oil immersion objective. The experimental setup used in the chemotaxis experiments are in Supplementary Figure S1.

### 2.4. Cell Culture

Jurkat cells were grown in ATCC-formulated RPMI-1640 Medium supplemented with 10% Fetal Bovine Serum (FBS) (Sigma-Aldrich, St. Louis, MO, USA) and 1% 100 U/ml penicillin-streptomycin (Sigma-Aldrich, MO, USA). Cells were sub-cultured every 3 to 4 days and kept in sterile incubation conditions for mammalian cells (37 °C, 5% CO_2_ and 90% humidity) according to ATCC protocols. Jurkat cells were assessed for expression of CD3, CXCR4 and CCR5 by flow cytometry (Figure S2). The percentage of CD3^+^ Jurkat cells was 27.8%. For the chemokine receptors, while 99.4% was CXCR4^+^, only 1.78% was found to be CCR5^+^. Although Jurkat cells originate from T cells where the CD3 molecule is constitutively expressed on the cell surface, their heterogeneity in terms of CD3 expression was previously reported [51]; we found no correlation between CD3^-^ and CXCR4^-^ expression.

For vital staining, 500 µL of cell suspension was collected and counted from the culture flask. The viability was evaluated using a Trypan Blue exclusion test with a hemocytometer. Samples with less than 90% viability were discarded prior to staining. Cells were then centrifuged and diluted to 1 million-cell/ml in 1× phosphate buffered saline (PBS) (Sigma-Aldrich). CellTracker Green BOPIDY dye (Thermo Fisher Scientific, Waltham, MA, USA) was added to the cell suspension to bring the suspension to a 25 µM final concentration (Figure S3). Cells were incubated for 45 min and subsequently centrifuged and diluted to 300,000 cells/ml in cell culture medium. Jurkat cells were introduced to the fibronectin coated gradient chambers by micropipetting 3 µL of cell/culture media through the cell introduction port. The final cell seeding density in the flow-free chambers was approximately 160 cells/mm^2^.

### 2.5. Image Acquisition and Cell Tracking

Confocal time-lapse sequences at 1-min intervals were captured 10 min after gradient establishment to track cells and observe gradients for 30 min (LASAF, Leica Microsystems, Wetzlar, Germany). Cells were then automatically tracked using the Linear Assignment Problem (LAP) algorithm [52] in the ImageJ plug-in TrackMate [53]. We selected 100 to 200 cell tracks from each chamber; tracks that lasted less than half of the imaging period were not considered (i.e., tracks that lasted less than 15 min for 30 min of observation) to reduce spurious cells that entered into or exited from the imaging region during the image acquisition. The tracking data were analyzed using the IBIDI Chemotaxis and Migration Tool (IBIDI GmbH, Martinsried, Germany), MATLAB (MathWorks Inc., MA, USA) and Numbers software (Apple Inc., CA, USA). Cells were initially sorted into motile and non-motile categories. Cells were considered non-motile if their accumulated displacement (e.g., path length) was less than 30 µm over 30 min of tracking (i.e., 1 µm/min average speed). Motile cells were subsequently analyzed for quantitative cell migration parameters.

The chemotactic motility of Jurkat cells was assayed using: (2) the percentage of motile cells (%); (3) the average migration speed (V) defined by the ratio of the accumulated migration distance ($d_{acc})$ to the time tracked (Δt); (4) the persistence of the migration track defined by the ratio of the net displacement ($d_{net}$) to the accumulated migration distance ($d_{acc})$ and (5) Chemotaxis Index (CI) defined by the ratio of the average distance traveled by the motile cells in the direction of chemokine gradient$\;(d_{dir})$ relative to the accumulated distance ($d_{acc}$) traveled by the motile cells [54].

(2) $$\%motile=\frac{n_{cells}\;(V<1\;µm/min)}{n_{cells}}\times 100$$

(3) $$V=\frac{d_{acc}}{\Delta t}\;$$

(4) $$Persistence=\frac{d_{net}}{d_{acc}}$$

(5) $$\;CI=\frac{d_{dir}}{d_{acc}}$$

Each experiment was repeated at least 3 times, and migration parameters were calculated for each experiment independently. A two-tailed unpaired student’s *t*-test was applied for statistical comparison of the data between two different conditions (* *p* < 0.05; ** *p* < 0.01; *** *p* < 0.001). The experiments with more than two conditions were analyzed using one-way ANOVA followed by post hoc two-tailed unpaired student’s *t*-test with Bonferroni correction for pairwise comparison. The cases where *p* > 0.05 were considered not significant (n.s.).

### 2.6. Finite Element Modeling

**Figure 3:**
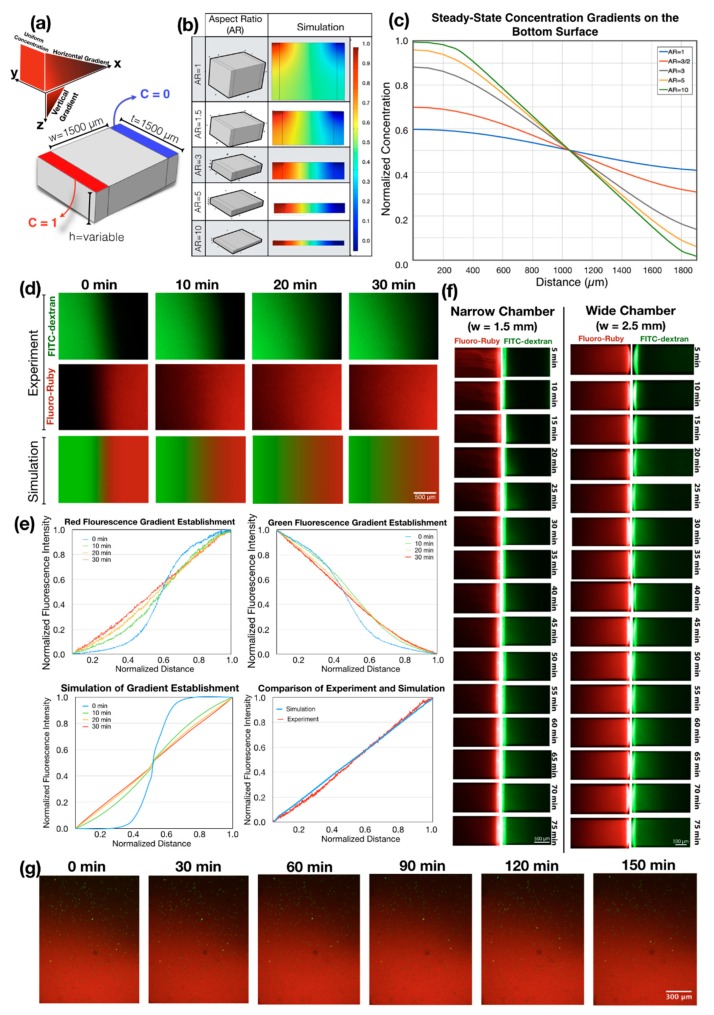
Generation of singular and dual competing concentration gradients in flow-free microfluidic chambers through coupled experimental and computational approaches. (**a**) A 3D model of gradient chamber with its dimensions and the boundary conditions used in simulations. (**b**) Results of simulations for controlling aspect ratios, which influences the gradients in the chambers. As the aspect ratio decreased, vertical concentration gradients began to dominate the system. (**c**) Concentration gradients measured at the bottom surface of the gradient chambers with controlled aspect ratios. As the aspect ratio decreased, the steepness of the gradients at the bottom surface of the gradient chamber decreased while maintaining the same average concentration. (**d**) Comparison of the experimental gradient generation and the simulation. The narrow gradient chamber was filled with FITC-Dextran (MW: 10,000) in half and Fluoro-Ruby (MW: 10,000) in the other half for faster generation of combinatorial gradients. Time-lapse images were captured every 10 min from both channels during the experimental gradient generation. (**e**) Comparison of the experiments and the simulations for the generation of transient and steady-state concentration gradient profiles. (**f**) Generation of linear concentration gradients across the flow-free gradient chambers without using the fast gradient generation technique. Unlike Figure 3d, here chambers were first filled with Fluoro-Ruby and FITC-Dextran was introduced later. The time required for the establishment of steady-state gradients was 4 times longer than the fluorescent reporters to diffuse through the entire gradient chambers. (**g**) Long-term observation of the linear concentration gradients in device with seeded cells. The concentration gradient was continuously monitored during the cell tracking experiments to confirm that the concentration gradients were within the specified range.

Gradient formation in flow-free chambers was simulated using the Transport of Diluted Species Module in COMSOL (Stockholm, Switzerland) to guide the design of the gradient chamber geometry. First, the flow-free chambers were generated as a rectangular solid with different aspect ratios. Inlet and outlet channels were represented by partitions along both sides of the chamber that defines regions with constant concentration. Boundary conditions were set along the partition surfaces to represent the sink and the source, respectively (C = 0 and C = 1; Figure 3a). Chambers were meshed with tetrahedral elements using the same mesh density (the maximum element size was 42 µm). Simulated concentration gradients were obtained along a line that traversed the chamber length on the bottom surface, representing the concentration gradient experienced by cells seeded onto the glass/fibronectin substrate in the flow-free gradient chamber.

### 2.7. Data Availability

All raw data, results and protocols related to this study are available upon request.

## 3. Results

### 3.1. Understanding Gradient Generation in a Flow-Free Microfluidic System through Numerical Simulation

Simulation of the concentration gradients generated in flow-free chambers revealed the formation of both vertical and horizontal gradients as the source and the sink surfaces were located only at the top of the gradient chambers. Cells seeded on the bottom surface of the gradient chamber were subject to gradients from both vertical and horizontal diffusion. The relative importance of the vertical gradient increased as the aspect ratio of the chambers (i.e., width/height) decreased. Vertical gradients created a decreasing concentration at the source side (C < 1), and an increasing concentration at the sink side (C > 0) (Figure 3a). Consequently, as the aspect ratio of the chamber decreases, the concentration gradient on the cells became shallower while maintaining the same average concentration. For example, in the gradient chambers with an aspect ratio of 1, the ideal normalized concentration gradient between C = 1 and C = 0 at the top surface became flatter, being C = 0.59 at the source side and C = 0.41 at the sink side of the bottom surface. An acceptable range of concentration change, where the gradient does not vary more than 5% from the ideal linear gradient at the bottom surface of the gradient chamber (e.g., being C = 0.96 and C = 0.04 at source and sink respectively) was achieved through an aspect ratio of 5 (Figure 3c). Thus, a flow-free chamber, which was able to produce a narrow gradient over 1500 µm in length, limited the height of the chamber to 300 µm. The concentration of the soluble factors used in the system should not have an effect on the time scale of the gradient generation nor on the steady-state gradient profiles based on equation 1. Therefore, the considerations above expressed in normalized values would be valid for different experimental conditions.

### 3.2. Experimental Concentration Gradient Generation

To demonstrate the generation of steady-state combinatorial concentration gradients in our flow-free gradient chambers, we introduced 50 µM of Fluoro-Ruby (MW = 10,000) to the flow channel on the right-hand side, and filled all of the gradient chambers. We then introduced 50 µM of FITC-Dextran (MW = 10,000) into the left flow channel and manually increased pressure on the left flow channel until the Fluoro-Ruby went back to the middle region of the chamber for equal filling of the two fluorophores in the chambers (Figure 3d, t = 0). Syringes were then connected to a double syringe pump to deliver both fluorescent reporters at a controlled volumetric flow rate of 0.2 µL/min. This flow rate continuously replenished the solution at the boundary regions as the fluorophores diffused across the flow-free gradient chambers. The time required for the formation of the steady-state gradient is defined as:

(6) $$t=\frac{L^{2}}{2D}$$

where L is the diffusion length and the D is the diffusion coefficient of the fluorophores. This approach allowed us to create combinatorial gradients and to reduce the time needed to equilibrate the gradient solely from the boundary sources by 75%. By using the diffusion coefficient for the 10 kDA FITC-Dextran of 1.33 x 10^−6^ cm^2^/s [55] the time required for the establishment of a linear gradients was calculated to be ~35 min for the narrow chamber and ~98 min for the wide chamber. The steady-state gradient we found using fluorescent reporters was similar to the simulated gradients (Figure 3d–f). In the cell migration experiments, we tracked the stability of the chemokine gradient by adding 50 µM Fluoro-Ruby to the chemokine solution in each experiment. Tracking cells and observing the chemokine gradient is important since the initial characterization of the gradient does not guarantee that these conditions will be maintained throughout each cell migration experiment.

One challenge with our approach was the bonding of the membrane filter to PDMS layers since non-uniform attachment or partial clogging could cause unstable concentration gradients. We found APTES-mediated membrane integration required a shorter time to fabricate (<30 min for complete assembly), had a higher success rate, and generated a stronger bond [56]. We tried another membrane integration technique that uses a thin PDMS prepolymer film as an intermediate adhesive layer [57,58]; however, the functional surface area of the PC membrane filter was frequently reduced, possibly due to the wetting. This approach caused partial clogging of the membrane by the PDMS prepolymer (Figure S4).

Finally, although this study mainly aimed to investigate cell motility at a population level, we confirmed the suitability of our microfluidic flow-free concentration gradient generation system combined with high resolution confocal microscopy for single cell migration studies (Figure S5).

### 3.3. Jurkat Cell Chemotaxis

#### 3.3.1. Increasing Substrate Fibronectin Concentrations Promote Random Cell Motility but Not Chemotaxis

We used our microfluidic approach to understand the effects of fibronectin concentration on the Jurkat cell motility. To do this, we coated the microfluidic gradient chambers with 0, 25, 500 and 1000 µg/mL concentration of fibronectin and assessed the effect of substrate fibronectin concentration on random cell migration and chemotaxis. We referred to 25 µg/mL and 1000 µg/mL as low substrate fibronectin concentration and high substrate fibronectin concentration, respectively. For cell migration experiments, we introduced cells labeled with CellTracker Green BODIPY dye to fibronectin-coated gradient chambers at a density of 300,000 cells/ml and placed the microfluidic system in a stage-top environmental chamber immediately after the seeding of cells. To establish the chemokine gradient over Jurkat cells, we introduced 100-nM CXCL12 to the inlet of the flow channels (Figure 3g), creating a concentration gradient with the slope of 66.6 nM/mm (plot of stable linear concentration gradient over time shown in Supplementary Figure S6).

**Figure 4:**
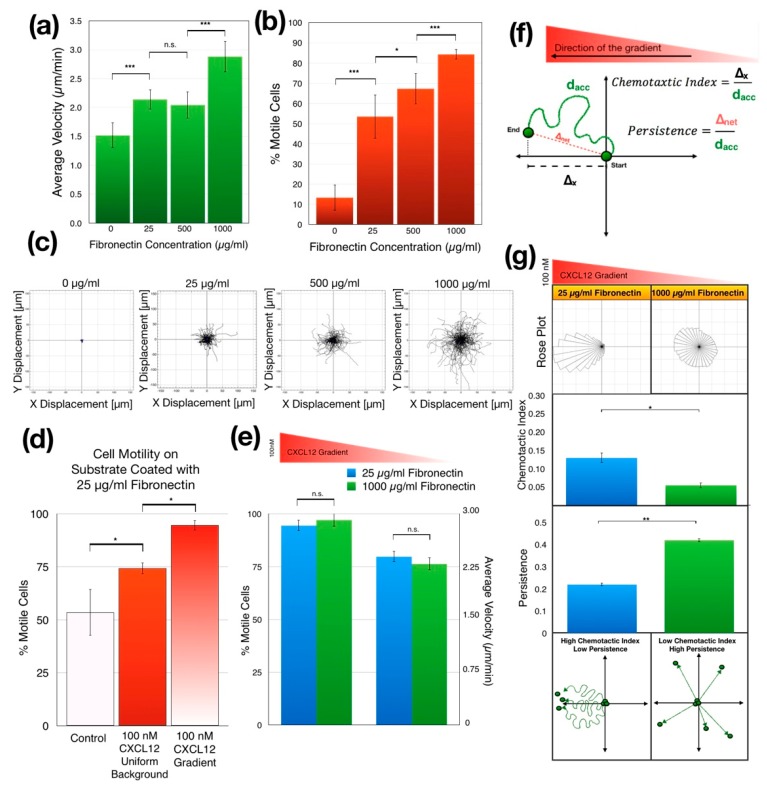
The effect of fibronectin concentration on Jurkat cell chemotaxis. (**a**) During random motion, the average speed of the migrating cells increased as the fibronectin concentration increased (*p* < 0.0001 by one-way ANOVA, ** *p* < 0.01, *** *p* < 0.001 by post hoc two-tailed unpaired student’s *t*-test with Bonferroni correction). (**b**) Also, during random motion, the percentage of motile cells increased as the fibronectin concentration increased (*p* < 0.0001 by one-way ANOVA, ** *p* < 0.01, *** *p* < 0.001 by post hoc two-tailed unpaired student’s *t*-test with Bonferroni correction). (**c**) Spider motility plots of randomly migrating Jurkat cells in microchambers coated with 0, 25, 500 and 1000 µg/mL of fibronectin, respectively. As the fibronectin concentration increased, cells spread more while maintaining their mass center at the origin (location of final mass center is shown as a blue dot). (**d**) Comparison of the percentage of motile cells in culture medium, in uniform 100-nM CXCL12 background and in 100-nM CXCL12 gradient in the microfluidic chambers coated with 25 µg/mL of fibronectin. (*p* < 0.0001 by one-way ANOVA, * *p* < 0.05 by post hoc two-tailed unpaired student’s *t*-test with Bonferroni correction). (**e**) In CXCL12 gradients, the motility of the cells appears to be independent of fibronectin concentration. Unlike Figure 4c,d, the change in fibronectin concentrations does not seem to alter the percentage of the motile cells nor the average migration speed when there is a chemokine gradient (*p* > 0.05 by two-tailed unpaired student’s *t*-test). (**f**) Schematic indicating our quantitative analysis approach for Jurkat cell motility in CXCL12 concentration gradient. (**g**) Jurkat cell chemotaxis in the gradient chamber coated with 25 µg/mL (referred to here as low concentration) and 1000 µg/mL (referred to here as high concentration) fibronectin. As the fibronectin concentration increased, the chemotactic index decreased (shown in the rose plots at the top) while the persistence increased (shown in the bar graphs at the middle). At high fibronectin concentrations Jurkat cells tend to migrate in a specific direction (high persistence), but this direction becomes independent of the direction of the chemokine gradient (low chemotactic index). This observation has been represented in the bottom schematics (** *p* < 0.01, * *p* < 0.05 by two-tailed unpaired student’s *t*-test). Data are presented as mean ± SD.

We first examined the effect of substrate fibronectin concentration on the random migration of Jurkat cells in the absence of a chemokine. As the fibronectin concentration used to coat the gradient chambers was increased from 0 to 1000 µg/mL, the average migration speed as well as the percentage of motile cells increased significantly (*p* = 2.21 × 10^−6^ for migration speed and *p* = 1.13 × 10^−9^ for the percent motility) while the overall Jurkat cell motility remained random (Figure 4a–c). Coating the microfluidic chambers with high fibronectin concentrations led to 8-fold increase in the percentage of motile cells compared to the cells migrating in non-coated chambers. In addition, there was 2-fold increase in the average velocity of the motile cells when the substrate was coated at a high fibronectin concentration.

Increasing surface fibronectin concentration gradually increased both the percentage of motile cells and their average velocity, however, this trend was disrupted once the chemokine was introduced. On the substrates coated with a low fibronectin concentration (25 µg/mL), addition uniform 100-nM CXCL12 background significantly increased the percentage of motile cells compared to the control lacking CXCL12 (*p* = 0.008). We then established a linear CXCL12 concentration gradient ranging from 0 to 100 nM across the microchannel. The presence of a concentration gradient further increased cell motility compared with a uniform CXCL12 concentration (*p* = 0.004) (Figure 4d). A statistically significant difference was not found for the percentage of motile cells and their average velocity between low and high fibronectin concentration when a chemokine gradient was present (Figure 4e). These results show that neither the fraction of motile cells nor their average migration speed were affected by the fibronectin concentration when CXCL12 chemokine gradient exists. In other words, while increasing fibronectin concentration promotes random cell migration, it does not increase the cell motility during chemotaxis.

#### 3.3.2. Increasing Substrate Fibronectin Concentration Creates Random Cell Migration Background during Chemotaxis

To better understand the role of the CXCL12 gradient and substrate fibronectin concentration in regulating chemotaxis, we tracked individual Jurkat cells seeded in microfluidic gradient chambers coated with either high or low fibronectin concentration as they migrated in a CXCL12 gradient, which ranged from 0 to 100 nM across the gradient chamber (gradient slope is 66.6 nM/mm). We calculated the CI and persistence for motile Jurkat cells using the tracking data. We found that the high surface fibronectin concentration significantly reduced the CI of the Jurkat cells (*p* = 0.014), increasing their random motion and decreasing their directedness as measured by CI (Figure 4f). At high fibronectin concentrations Jurkat cells migrated more uniformly in all directions regardless of the direction of the chemokine gradient (Figure 4g, top left). Interestingly, while the CI of their motion towards the CXCL12 gradient decreased, their persistence significantly increased with increasing fibronectin concentration (*p* = 0.045) (Figure 4g, middle). In other words, as the surface fibronectin concentration increased, Jurkat cells migrated more persistently in random directions independent of cues from the chemokine gradient (Figure 4g, bottom).

#### 3.3.3. Jurkat Cells Migrate Faster in Shallower CXCL12 Gradient

**Figure 5:**
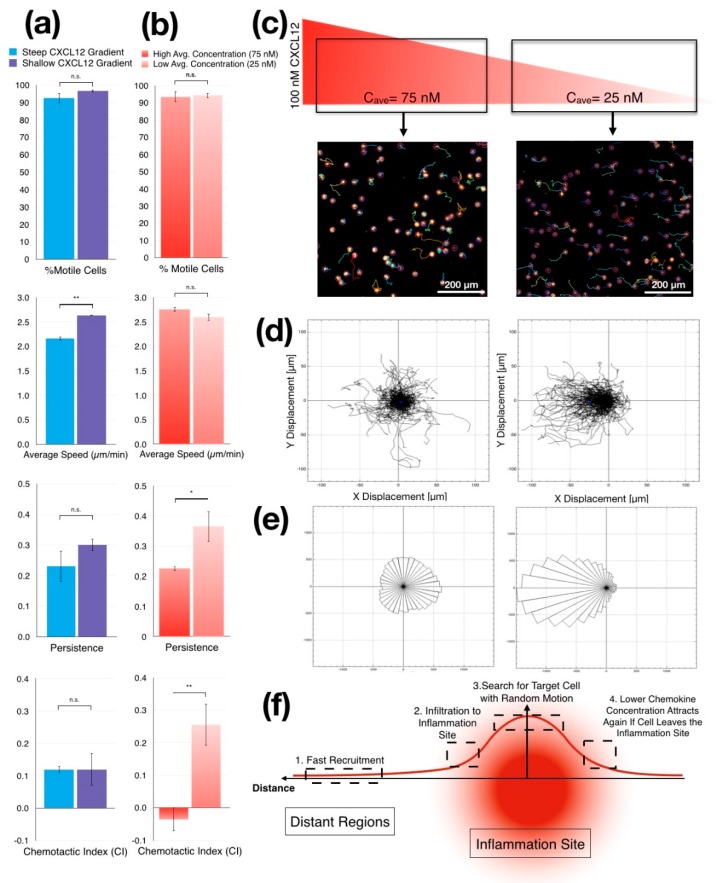
Chemotaxis of Jurkat cells in different CXCL12 gradient modalities (**a**) Quantitative analysis of cell movement shows differences between migration parameters from cells that migrate in shallow CXCL12 gradient (wide chamber, 40 nM/mm) and steep CXCL12 gradient (narrow chamber, 66 nM/mm). Although the increased steepness does not alter the motile cell ratio and chemotactic index, it slows the average speed and persistence of the migration (** *p* < 0.01 by two-tailed unpaired student’s *t*-test). (**b**) Quantitative analysis shows that average CXCL12 concentration strongly affects the persistence and the chemotactic index of the migrating Jurkat cells, while it does not alter the percentage of motile cells or the average speed of the migrating cells (** *p* < 0.01, * *p* < 0.05 by two-tailed unpaired student’s *t*-test). (**c**) We examine our wide gradient chamber by dividing it into two regions with different average chemokine concentrations while maintaining the same gradient steepness. While the region on the left has an average concentration of 75-nM CXCL12, the region on the right has an average of 25-nM CXCL12. (**d**) Spider motility plots of the Jurkat cell chemotaxis in two sub-regions of the wide gradient chamber. While the cells at the higher average CXCL12 concentration (left) appear to be migrating randomly, the ones in the lower average CXCL12 concentration (right) appear to migrate toward the higher concentration of CXCL12 in the gradient. (**e**) Rose plots show the difference between two subregions of the wide gradient chamber in terms of the spreading of the Jurkat cells. Although, Jurkat cells which were spread equally in all the directions at a high average CXCL12 concentration, show a random migration pattern, they migrated towards the CXCL12 gradient in the right side of the chamber. (**f**) Schematics of a potential description of the accumulation and random motility of immune cells in the inflammation zone related to high chemokine concentrations that may function as a chemical trap. Data are presented as mean ± SD.

To assess cellular responses to controlled gradient slopes, we compared chemotactic behaviors of Jurkat cells in narrow and wide chambers where we created 2 independent chemokine gradients with different gradient steepness but with the same average chemokine concentration. As previously described, we filled the source channel with 100-nM CXCL12, diluted in cell culture medium while the culture medium alone was supplied to the sink channel to create gradients of CXCL12 with different slopes simultaneously (66.6 nM/mm in the narrow chamber and 40 nM/mm in the wide chamber). We tracked cells in both gradient chambers in parallel and calculated their migration parameters (Figure 5a). We coated gradient chambers with 25 µg/mL fibronectin to enable Jurkat cell migration and allow them to maintain a directional response to a chemokine gradient. The slope of the gradient did not significantly alter the percentage of motile cells as more than 90% of cells were motile in both chambers. As expected, CI was also similar in both chambers. On the other hand, we noted substantial increase in the persistence in a shallow CXCL12 gradient, but this increase was not significant (Figure 5a). Surprisingly, we found the average migration speed of the motile Jurkat cells increased significantly when the slope of CXCL12 gradient was lowered to 40 nM/mm (*p* = 0.008).

#### 3.3.4. Jurkat Cells Migrate Randomly in CXCL12 Gradients with High Average Concentration

To test whether Jurkat cells were responding to cues in the gradient or merely differing amounts of CXCL12, we investigated the migratory response of Jurkat cells in the same chamber but at different average concentrations of CXCL12. In the wide gradient chamber, we tracked the Jurkat cells closer to the source channel and the ones closer to the sink channel separately, based on their initial positions within the chamber. We then compared the migratory behavior of Jurkat cells close to the source channel with cells that were closer to the sink channel. Cells in both subgroups had the same gradient slope (40 nM/mm) as the gradient was linear across the chamber, but the first group was in the region of the chamber where the average CXCL12 concentration was 75 nM (high average concentration), and the other subgroup was in the region of the chamber where the average CXCL12 concentration was 25 nM (low average concentration). The change in the average CXCL12 concentration did not cause a significant alteration in the percentage or the average migration speed of the motile cells (Figure 5b). This result was similar to the results presented above with the different surface fibronectin concentrations (Figure 5b vs. Figure 4e). However, the chemotactic behaviors of the Jurkat cells in different subgroups were significantly different (Figure 5c). While Jurkat cells in the low average CXCL12 concentration were migrating towards the CXCL12 gradient with high persistence and CI, those in the high average CXCL12 concentration migrated away from the CXCL12 concentration with significantly lower persistence (*p* = 0.014) and CI (0.0018). This finding suggested a migratory behavior closer to the random motion observed with uniform CXCL12 background (Figure 5c,d). Nonetheless, substantially higher numbers of Jurkat cells were motile, showing that cells still distinguished between a uniform chemokine background and a chemokine gradient. This suggests that CXCL12 saturate chemotactic responses of Jurkat cells at concentrations above 75 nM in a manner similar to the saturation of T cells responses to CCL19 or CCL21 concentrations above 100 nM [59,60]. Also, using a different microfluidic system, Jurkat cells were shown to have significantly reduced chemotactic response to a CCL19 gradient when the CCL19 concentration was reduced from 100 nM to 10 nM while preserving their average migration speed [61] similar to our chemotaxis results. Overall, these observations and our results suggest that both the concentration gradient and the average concentration of the chemokines directs the chemotactic behavior of different immune cells and that both Jurkat cells and T cells share saturable motility responses to chemotactic cues albeit to different chemokines.

## 4. Discussion

The profile of the steady-state gradients within the microfluidic flow-free gradient chambers is highly related to the aspect ratio of the chambers. If the profile is not taken into account, the actual chemokine gradient presented to the cells in the chambers can be significantly different from the boundary values defined at the flow channels. We generated steady-state concentration gradients in these 3D-printed microfluidic chambers within 35 min using 10 kDa FITC-Dextran as a fluorescent probe. The diffusion coefficient of FITC-Dextran (D = 1.33 x 10^−6^ cm^2^/s) is lower compared to the other fluorescence probes with similar molecular weight (i.e., D = 4.34 x 10^−6^ cm^2^/s for Alexa 488 (MW: 10,000)). Using these fluorescent probes, it is possible to demonstrate the establishment of gradient in the same microfluidic system three to four times faster. However, we used a fluorescent probe with a lower diffusion coefficient to be conservative in showing that the steady-state concentration gradient of the actual chemokine was established when we started tracking the cells. It is essential for the functional regions of membrane to remain intact to maintain stable gradients over time. Since diffusion only occurs across a small portion of the membrane surface, partial or complete clogging of the membrane can result in unstable gradients [49,62]. Therefore, membrane clogging can critically limit applications in systems like ours where only a small region of a membrane is effectively used. For this reason, we tested multiple membrane processing techniques and found that APTES-mediated membrane integration was the most effective technique to create a strong bond without clogging (Figure S3).

CXCL12 and fibronectin independently regulate Jurkat cell motility. In a uniform concentration of 100-nM CXCL12 (no gradient) more Jurkat cells showed motility compared to controls lacking CXCL12. Interestingly, even more cells migrated in the gradient of 100-nM CXCL12 (Figure 4d). Therefore, CXCL12 appears to have two distinct functions. First, uniform levels of CXCL12 activate cells to became motile. Second, a gradient of CXCL12 provide an additional cue for directional chemotactic response. A very similar gradual increase in the Jurkat motility has been previously observed with a uniform CCL2 background and CCL2 gradient using conventional Boyden chambers [63]. Although, we have shown that the fibronectin concentration can regulate the extent of random motility of the Jurkat cells, we did not see a significant difference in the percentage of motile cells or their average velocities with 100-nM CXCL12 gradient in chambers coated with low (25 µg/mL) or high fibronectin concentrations (1000 µg/mL) (Figure 4a,b vs. Figure 4e). In a potentially similar manner, neutrophils and in-vitro activated T cells have also been shown to have constant chemotaxis speeds regardless of the extracellular matrix concentration [64] or the type of chemokine [65] while having as much as two times higher average migration speed than Jurkat cells [66].

The addition to the chemokine gradient, the surface-bound fibronectin concentration also regulates the random motility as well as the chemotactic behavior of Jurkat cells. Jurkat cells within a chemokine gradient migrate in all directions with high persistence regardless of the direction of the chemokine gradient when fibronectin concentration is high, creating a migratory pattern similar to random migration (Figure 4g). This finding parallels previous studies where the increased fibronectin concentration has also been reported to increase random migration in chemotactic cells [64,67]. On the other hand, in the very same chemokine gradient, cells that were seeded in gradient chambers coated with low fibronectin concentrations show opposite behavior by migrating in the direction of the chemokine gradient with high CI and low persistence. The decoupling of the Jurkat cell motility and directionality has been shown to be related to PTEN-modulated actin polymerization [68]. These results suggest that both extracellular matrix (ECM) concentration and chemokine gradients in the cellular microenvironment regulate chemotactic responses of Jurkat cells in an interactive manner.

Distinctive migratory responses in different chemokine gradients suggest that chemokine gradients may regulate the spatial bounds of the immune response. During a local inflammation in a defined region in the body, immune cells are recruited rapidly to the inflammation site through the orchestration of chemokines released from the site of inflammation [69]. Since these chemokines diffuse in all directions in 3D, they create nonlinear concentration gradients where the slope gradient is high at the regions proximal to the inflammation site and becomes shallower at the distant regions [70] (Figure 5f). Therefore, immune cells that reside at the distant regions of the body should initially sense these shallow chemokine gradients and respond by moving rapidly toward the inflammation site [71] (Figure 5f.1). Remarkably, first responders of the immune system are recruited from distant regions to injury sites within hours [72]. Our experimental data with Jurkat cells as a model cell line also show faster directional migration in shallower CXCL12 gradient supporting this concept.

The rapid chemotactic response at shallower gradients, where differential receptor occupancy across the cell membrane [73] would be expected to be less significant, may be explained by an alternative chemotaxis concept [74]. After recruitment (Figure 5f.2), activated immune cells accumulate at the inflammation site [75] but require cell–cell contact to carry out their effector function [76]. Thus, immune cells would need both a general attraction to the site of inflammation but also would require specific cues that allows them to target specific cells within the infected tissue. The strong chemotactic response that we have observed in this study at low chemokine concentrations accompanied with a chemokinetic response at high chemokine concentrations appears to support this two-stage scenario. One way to consider this is in a “chase” and “search” mode where the response of the cells depends on the average concentration of the chemoattractant. In "chase" mode, immune cells approach the inflammation site following long-range directional cues from chemokine gradients. Once in proximity of infected cells, immune cells respond to high concentrations of chemokines engage in a "search" mode, moving rapidly but with low persistence, to reach their targets (Figure 5f.3). If the immune cell leaves the infected tissue as a result of this random motion, the average chemokine concentration decreased again which would again start the migration through the chemokine gradient, resulting in the cells returning to the inflammation site (Figure 5f.4). These dual modes of chemokine functions would allow immune cells to accumulate within the infected tissues without blocking them from performing a local search for their infected targets with highly motile random migration. Our experimental data with Jurkat cells as a model for T cells mimics these motility behaviors as shown with quantified live cell tracking analysis.

## 5. Conclusions

We developed a flow-free gradient chamber within a microfluidic system that enables the generation and maintenance of single or combinatorial concentration gradients. The wide gradient chamber of our enhanced system allows population-wide cell migration assays with single cell sensitivity using high resolution microscopy. We constructed the system using additive manufacturing and replication molding techniques, demonstrating the feasibility and speed of fabrication, even in the absence of specialized microfabrication equipment and sophisticated facilities. The resulting spatiotemporally controlled fluidic environments were used to induce cellular responses to different gradient profiles in chambers with different aspect ratios. The transient and steady-state gradient profiles obtained in experiments agreed with theoretical predictions and simulation results.

In addition, in applying the flow-free gradients of CXCL12, we demonstrated Jurkat cell chemotaxis in response to different concentration gradients and the coupled functionality of these gradients with surface fibronectin concentration. Our analysis of Jurkat cell chemotaxis to CXCL12 gradients in the context of different fibronectin concentrations demonstrated that fibronectin increased the percentage of motile cells and the average speed of random cell motion, but did not alter these properties when there was a chemokine gradient. We also found that fibronectin significantly altered the CI and the persistence of the motile cells during chemotaxis. This system also allowed us to show that high concentrations of CXCL12 saturated the Jurkat cells, resulting in random migration even in the context of a chemokine gradient. The dual mode of response to chemokines proposed in our microfluidic experiments mimics the overall immune cell motility in vivo. Our novel technique facilitates further studies of the relationship between different cellular microenvironments and chemotactic responses, which are relevant to a wide range of applications from immunotherapy to microfluidics technologies.

## Supplementary Materials

The following are available online at https://www.mdpi.com/2072-666X/11/4/384/s1, Figure S1: Experimental setup for Jurkat cell chemotaxis experiments. Figure S2: Receptor expression analysis data for Jurkat cells. Figure S3: Fluorescent images of Jurkat cells stained with different concentration of CellTracker Green BOPIDY. Figure S4: Comparison of two different membrane integration techniques. Figure S5: High resolution images of Jurkat cells stained with CellTracker Green BOPIDY in flow-free microfluidic gradient chamber.
