# Supplementary material for: Chemotactic Responses of Jurkat Cells in Microfluidic Flow-Free Gradient Chambers
Source: Micromachines (Basel). 2020 Apr 4;11(4):384. doi: 10.3390/mi11040384 (PMC7231302; doi:10.3390/mi11040384)
Supplement: Supplementary file 1 [file micromachines-11-00384-s001.pdf]

micromachines

Supplementary Materials: Chemotactic Responses of Jurkat Cells in Microfluidic Flow-Free Gradient Chambers

Utku M. Sonmez, Adam Wood, Kyle Justus, Weijian Jiang, Fatima Syed-Picard, Philip R. LeDuc, Pawel Kalinski and Lance A. Davidson

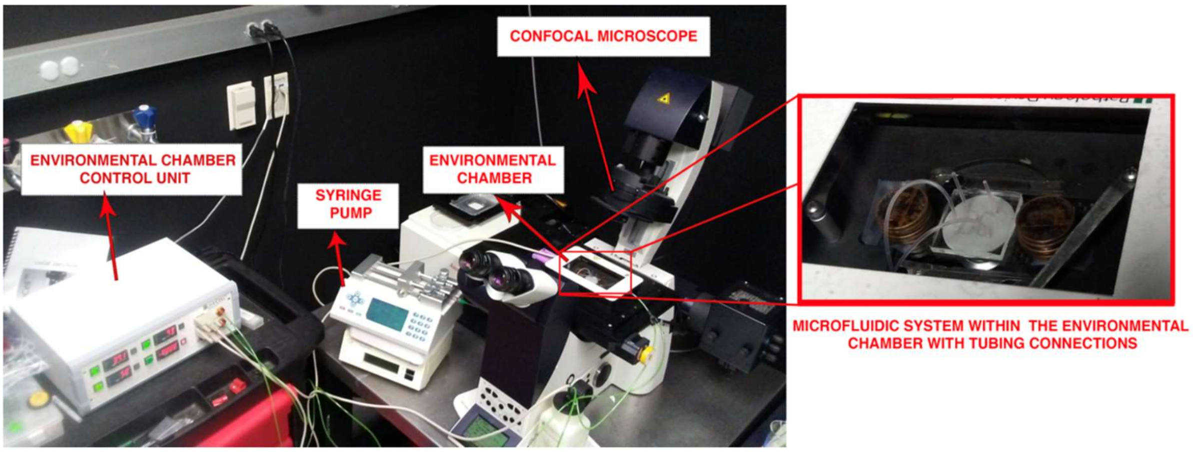

Figure S1. Our experimental setup for Jurkat cell chemotaxis experiments. The microfluidic device was put in stage-top environmental chamber to maintain incubation conditions (37 °C, 5% CO_2, and 90% humidity). A syringe pump was connected to the microfluidic device to replenish the chemokine solution at a very low flow rate. Imaging was accomplished using a Leica SP5 confocal microscope.

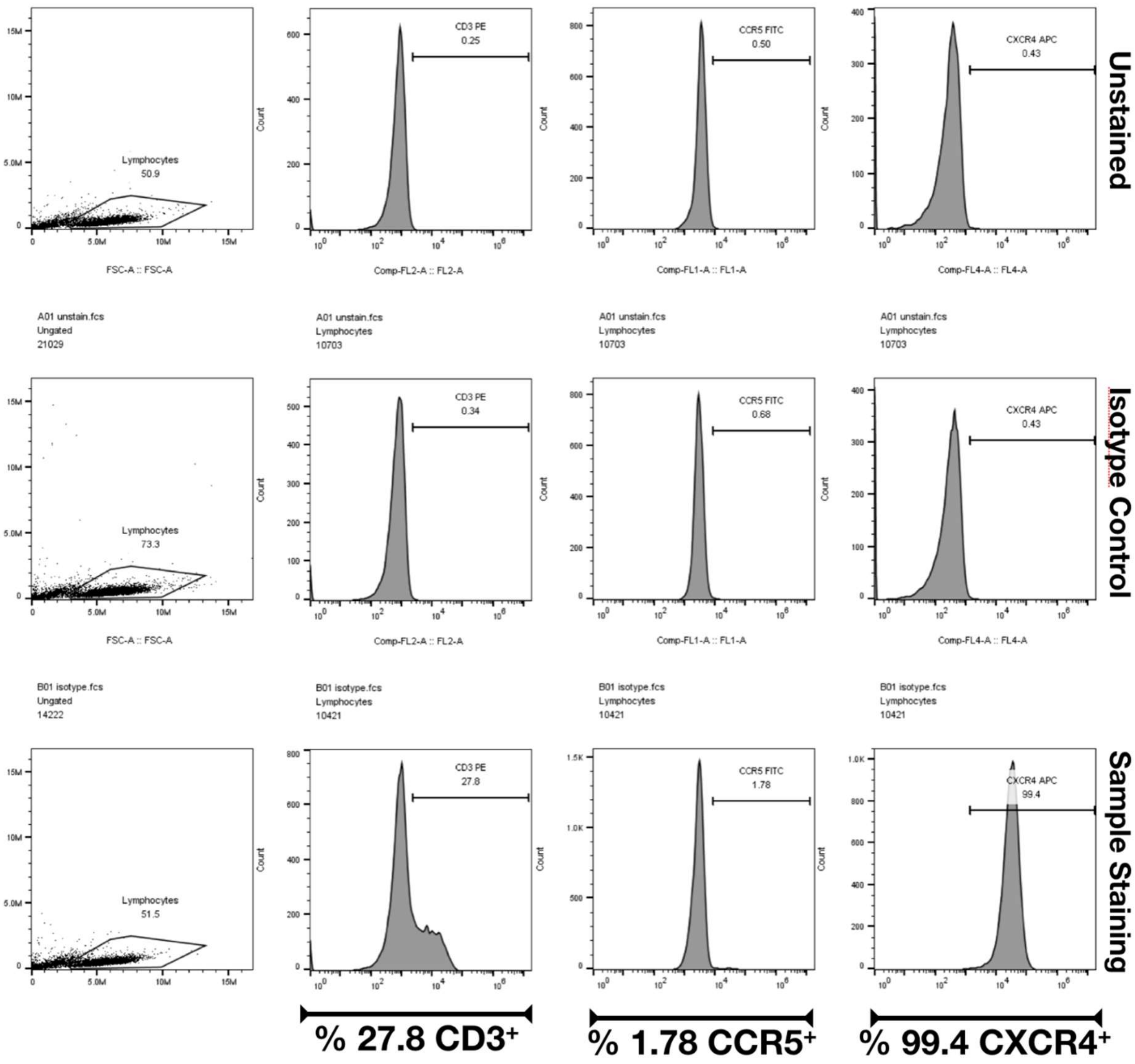

Figure S2. Receptor expression analysis data for Jurkat cells.

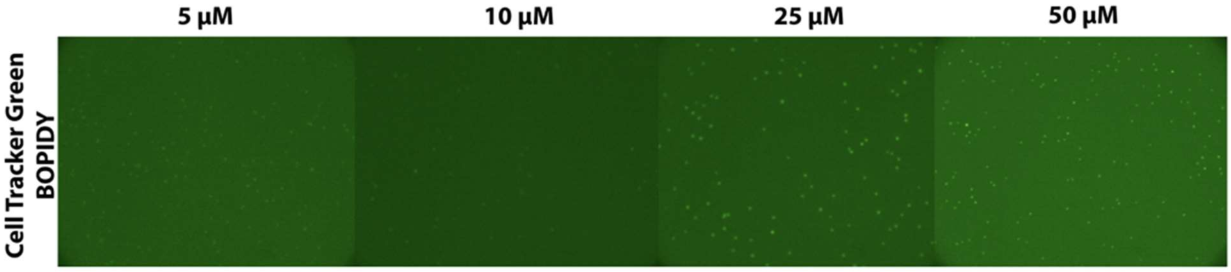

Figure S3. Fluorescent images of Jurkat cells stained with different concentration of CellTracker Green BOPIDY. We did a range of tests for examining the concentration of CellTracker for use in our studies. Two dye concentrations (25 and 50 μM) results in better imaging and 25 μM concentration was selected for staining to maintain the image quality while minimizing the necessary dye concentration.

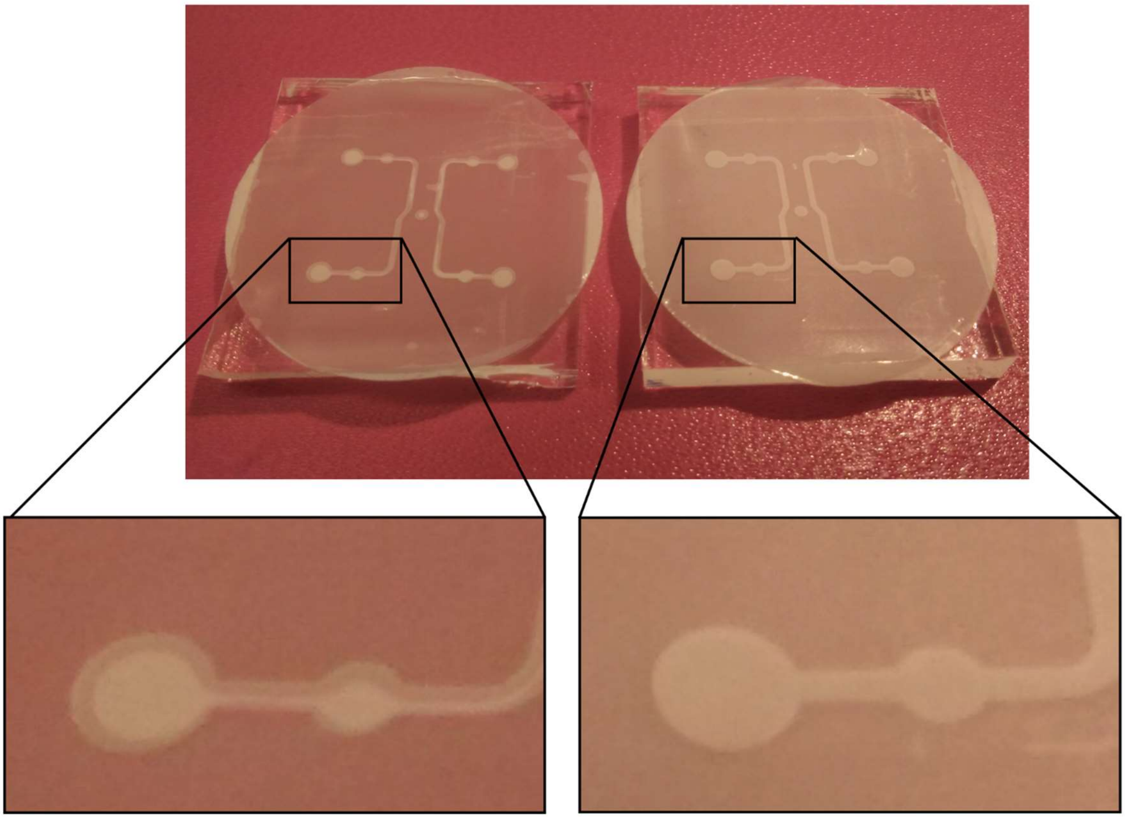

Figure S4. Comparison of two different membrane integration techniques. (left) A PC membrane is integrated to a microfluidic channel using PDMS prepolymer as an intermediate adhesive layer. In short, PDMS was mixed with its curing agent with 10:1 ratio. Later, the PDMS prepolymer was diluted in Toluene with 1:1 mass ratio. The diluted PDMS prepolymer was poured on a pre-cleaned glass slide, and spun 3 s at 500 rpm and 60 s at 1500 rpm, respectively, to obtain a 4 μm thick layer of PDMS prepolymer on the glass slide [1]. A coated glass slide was then stamped against the patterned surface of the PDMS microchannel. Finally, the membrane was brought in contact with the microchannel for bonding. Assembly was maintained at 60 °C in an oven for 2 hours for the curing of the PDMS prepolymer to induce irreversible bonding. (right) A PC membrane integrated to a microfluidic channel with APTES treatment and subsequent oxygen plasma bonding (details of the fabrication are in the main text). Unlike the first technique, here the membrane was bonded to the PDMS with covalent bonding to ensure that only the region of the membrane that is in contact with the PDMS was bonded while the rest of the membrane remained intact. However, in the first technique, the diluted PDMS prepolymer was absorbed by the membrane, partially sealing some regions of the membrane that were supposed to remain intact (see the insets). This resulted in a narrowed effective membrane region which affected adversely the gradient generation mechanism. Furthermore, the intermediate PDMS prepolymer layer required very careful handling of the membrane since contamination of membrane with the PDMS prepolymer could lead to irreversible clogging of the membrane. These issues caused a low success rate, especially during the alignment of the two PDMS layers where any relative motion between two layers resulted in membrane clogging. Finally, while the integration of the membrane to the PDMS layer occurred instantaneously in the APTES technique, it required subsequent curing steps of the assembly in the adhesive PDMS prepolymer technique which, in turn, increased total fabrication time.

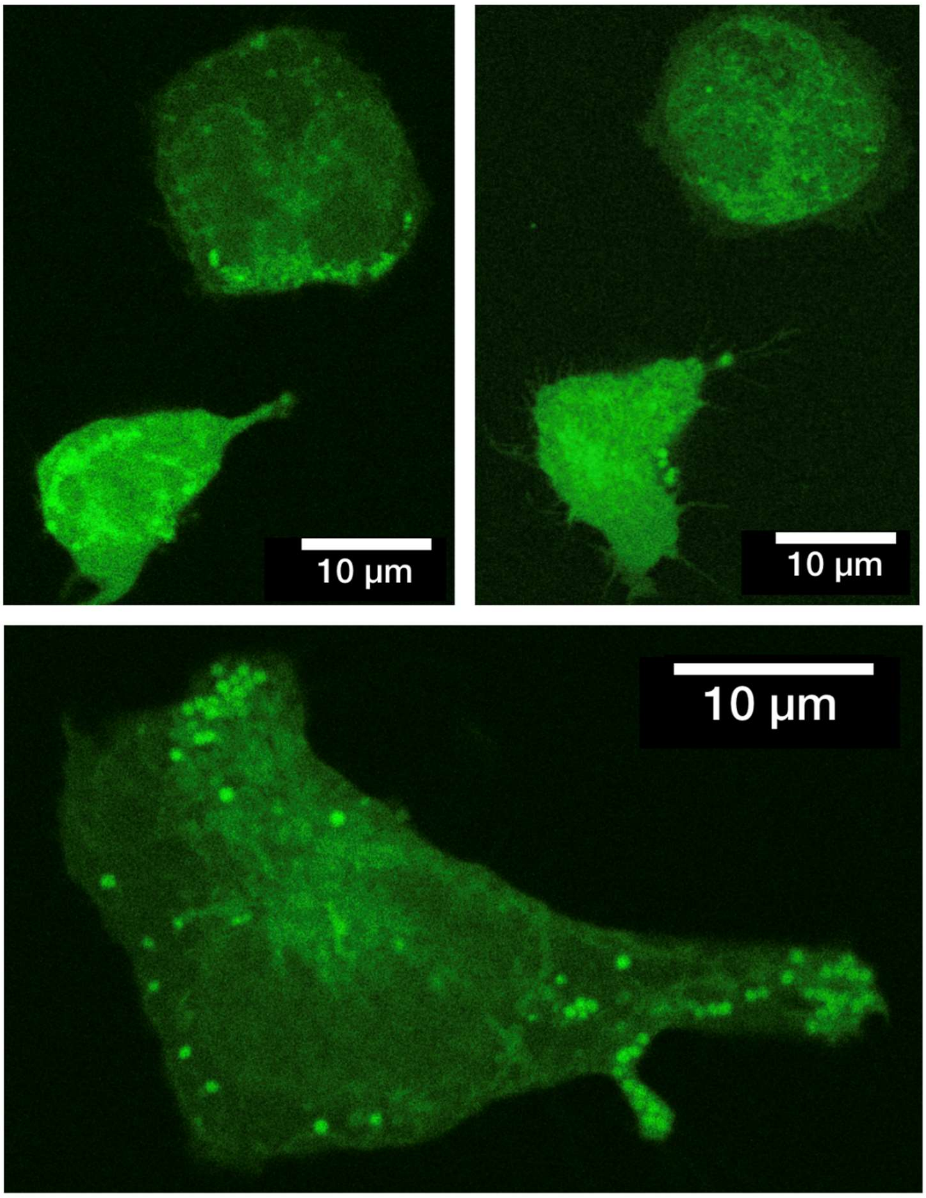

Figure S5. High resolution images (63×) of Jurkat cells stained with CellTracker Green BOPIDY in flow-free microfluidic gradient chamber.

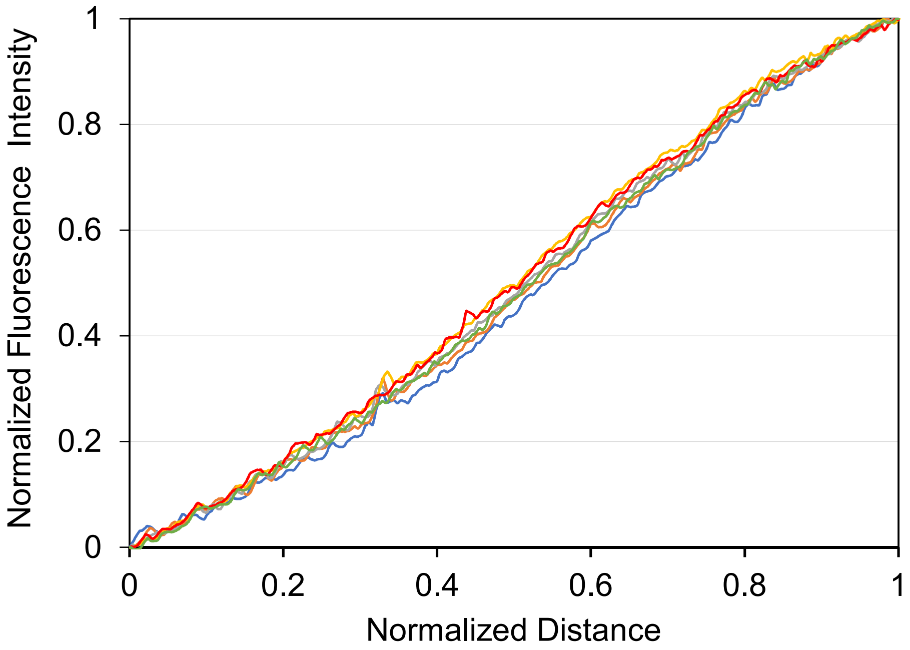

Figure S6. Plot displaying stable linear concentration gradient over time in flow-free chamber (blue line = 0 min.; orange line = 30 min.; grey line = 60 min.; yellow line = 90 min.; red line = 120 min.; green line = 150 min.).

Reference

1. Wu, H.; Huang, B.; Zare, R.N. Construction of microfluidic chips using polydimethylsiloxane for adhesive bonding. *Lab Chip* **2005**, *5*, 1393–1398.

© 2020 by the authors. Submitted for possible open access publication under the terms and conditions of the Creative Commons Attribution (CC BY) license (http://creativecommons.org/licenses/by/4.0/).
